# Hippocampus and Parahippocampus Volume Reduction Associated With Impaired Olfactory Abilities in Subjects Without Evidence of Cognitive Decline

**DOI:** 10.3389/fnhum.2020.556519

**Published:** 2020-09-30

**Authors:** Satomi Kubota, Yuri Masaoka, Haruko Sugiyama, Masaki Yoshida, Akira Yoshikawa, Nobuyoshi Koiwa, Motoyasu Honma, Ryuta Kinno, Keiko Watanabe, Natsuko Iizuka, Masahiro Ida, Kenjiro Ono, Masahiko Izumizaki

**Affiliations:** ^1^Department of Physiology, Showa University School of Medicine, Tokyo, Japan; ^2^Division of Neurology, Department of Medicine, Showa University School of Medicine, Tokyo, Japan; ^3^Sensory Science Research, Kao Corporation, Tokyo, Japan; ^4^Department of Ophthalmology, Jikei Medical University, Tokyo, Japan; ^5^Department of Health and Science, University of Human Arts and Sciences, Saitama, Japan; ^6^National Hospital Organization Mito Medical Center, Ibaraki, Japan

**Keywords:** morphological brain changes, olfaction, recognition, parahippocampus, orbitofrontal cortex, amygdala, hippocampus

## Abstract

The aim of this study was to investigate the relationship between olfactory recognition and morphological changes in olfactory brain regions including the amygdala, hippocampus, rectus, parahippocampus, orbitofrontal cortex, and medial frontal cortex in 27 elderly subjects and 27 younger healthy controls. The specific aim of the study was to determine which brain areas are associated with the initial decline of olfaction in elderly subjects, which occurs before the onset of dementia. All subjects underwent magnetic resonance imaging to measure anatomical brain volume and cortical thickness, and subjects were assessed using tests of olfactory acuity and cognitive function measured with the Montreal Cognitive Assessment. Overall brain volume reductions were observed in elderly subjects compared with young healthy controls, but only reduction in the volume of the left hippocampus was associated with decreased olfactory ability. The parahippocampus of elderly subjects was not different from that of controls; the extent of the reduction of parahippocampus volume varied among individuals, and reduction in this region was associated with olfactory decline. Similarly, parahippocampus thinning was associated with decreased olfactory function. The path analysis showed direct and indirect effects of hippocampus and parahippocampus volume on olfactory ability and that volume reductions in these areas were not associated with cognitive function. Parahippocampus volume reduction and thinning exhibited individual variation; this may be the first appearance of pathological changes and may lead to dysfunction in the connection of olfactory memory to the neocortex. Parahippocampus change may reflect the first sign of olfactory impairment prior to pathological changes in the hippocampus, amygdala and orbitofrontal cortex.

## Introduction

Olfaction is a unique sensory modality. Odor information ascends to the primary olfactory area, the piriform cortex, and other areas, including the entorhinal cortex (ENT) and amygdala (AMG), without passing through the thalamus (Sobel et al., 2000). The olfactory information then engages memory retrieval and emotional reaction mediated by the hippocampus (HI) and AMG. Finally, all information converges in the orbitofrontal cortex (OFC). The OFC plays a role in identification of the odor and labeling of the associated emotions (Rolls, 2001).

The neural network linking the olfactory limbic areas and the frontal regions is sustained by the ENT and parahippocampus (para-HI) in processing odor memory retrieval and applying emotional context to subjective recognition (Naya, 2016). Processing of olfactory perception occurs if all functional areas and connectivities are intact; however, declining olfactory ability or olfactory impairment is present in mild cognitive impairment (MCI) (Roberts et al., 2016) and neurodegenerative disorders and may be caused by abnormalities in olfactory brain regions (Doty et al., 1988; Hawkes, 2003).

Impaired olfactory ability might be caused by three factors. First, it might be caused by brain abnormalities in the AMG and HI. In Alzheimer’s disease (AD) and Parkinson’s disease, pathological changes, including accumulation of senile plaques and neurofibrillary tangles, occur first in the HI and AMG (Mesholam et al., 1998), which are two key areas for olfaction. This may explain why olfactory impairment is often observed as the first sign of these neurodegenerative disorders, prior to the typical symptoms (Doty et al., 1988; Mesholam et al., 1998; Hawkes, 2003). Second, impairments might be attributed to pathological changes in the para-HI, which are often reported in AD patients (Koychev et al., 2017). Third, decline of frontal cortex function has also been reported in these patients (Franzmeier et al., 2017; Chung et al., 2019). It is possible that loss of olfactory recognition might be caused by a decrease in frontal cortex function, such as the OFC.

In this study, we determined which olfactory brain areas were associated with the initial decline of olfactory ability in elderly subjects. To identify how olfactory brain volume in elderly subjects differed from that in younger subjects, we investigated olfaction ability and related brain volumes in older subjects and younger control subjects. Comparisons between these two groups might be helpful to clarify specific features of olfactory alteration in the brain. The HI, AMG, para-HI, OFC, rectus, and medial prefrontal cortex (MFC) were examined as regions of interest (ROI) based on a previous report (Watanabe et al., 2018). HI volume reduction has been reported in numerous conditions (Geuze et al., 2005), and volume reduction in the HI and AMG has been associated with olfactory recognition ability (Masaoka et al., 2020). In this study, we focused on the association between olfactory ability and volume measurements; however, other reports have found that cortical thickness changes were closely related to volume changes (Storsve et al., 2014). Exploratory analysis was performed for cortical thickness measurements of the para-HI, OFC, rectus, and medial frontal regions, and we determined whether cortical thinning contributes to volume changes as well as olfactory ability.

## Materials and Methods

### Participants

Initially, 30 elderly subjects (mean age 73.7 ± 5.5 years), living independently and with self-reported absence of memory and mild cognitive deficits, participated in the study. Subjects were recruited from Meguro Human Resource Center, Tokyo. Twenty-seven younger healthy controls (HCs; mean age 37.8 ± 9.1 years), recruited from graduate students and university staff, were also tested. The exclusion criteria for all participants were (1) a history of head injury or seizures and (2) diagnosis of a neurological disorder. Three elderly subjects were excluded because of previous cerebral infarction (2 subjects) or subarachnoid hemorrhage (1 subject), resulting in a final total of 27 elderly subjects. The demographic characteristics of the 27 elderly subjects and young HCs, including age, sex, height, dominant hand and duration of education, are summarized in Table 1. All experiments were conducted in accordance with the Declaration of Helsinki^1^. The study was approved by the Ethical Committees of Showa University School of Medicine, and all participants provided written informed consent prior to participation.

**TABLE 1:** Demographic data of young healthy controls and elderly subjects.

	Younger HC (24–55)	Elderly subjects (62–84)
Total (M/F), No.	27 (Female, 10/Male, 17)	27 (Female, 14/Male, 13)
Age, y	37.8 ± 9.1	73.7 ± 5.4***
Handness (R/L)	(Right, 26, Left, 1)	(Right, 26, Left, 1)
Years of education	17.8 ± 2.9	13.8 ± 2.5***
MoCA-J	28.6 ± 2	25.5 ± 2.2***
Olfactory detection	−0.4 ± 0.7	0.9 ± 0.7***
Olfactory recognition	0.2 ± 0.7	2.3 ± 0.6***
		(5 subjects with olfactory impairment)
ICV	1570523 ± 1124190	1454411 ± 153196*
WBV	1157413 ± 81519	990973 ± 91716***
L-HI	4023 ± 282	3472 ± 349***
R-HI	4261 ± 360	3666 ± 443**
L-AMG	1731 ± 176	1388 ± 228**
R-AMG	1795 ± 171	1527 ± 214**
L-OFC	5939 ± 674	4921 ± 613***
R-OFC	6672 ± 670	5811 ± 402**
L-paraHI	3350 ± 596	3096 ± 496
R-paraHI	3590 ± 559	3129 ± 510
L-rectus	2271 ± 320	2151 ± 244
R-rectus	2015 ± 288	1753 ± 147
L-MFC	10288 ± 1539	8214 ± 1280**
R-MFC	9309 ± 1307	7495 ± 814***

***P < 0.0001, **P < 0.001, *P < 0.01.

### Olfactory Ability and Cognitive Assessment

Odor detection acuity and odor recognition acuity were evaluated in all subjects using the T&T olfaction test (Takasuna Co., Ltd., Tokyo, Japan). The T&T test is well correlated with the University of Pennsylvania Smell Identification Test (Kondo et al., 1988). Details of the olfaction test have been described elsewhere (Masaoka et al., 2005). In brief, the test is conducted with five odors (odor A, β-phenyl ethyl alcohol; odor B, methyl cyclopentenolone; odor C, iso-valeric acid; odor D, γ-undecalactone; odor E, skatole). Each odorant is presented, dissolved in propylene glycol, at eight different concentrations, each 10 times more concentrated than the last; concentrations are labeled from −2 to + 5. The five odors are presented randomly but at the same concentration in each trial. The trials begin with the lowest concentration and are repeated with progressively higher concentrations. During each trial, the subject is asked whether an odor was perceived. The concentration at which the odor is perceived but not identified is considered the “detection level.” As the concentration increases, the subject is able to identify the odor. The subject is required to identify each odor and describe the kind of odor. The concentration at which an odor is first identified is considered the “recognition level.” Each subject’s odor detection threshold is expressed as the average of all odor threshold scores (A + B + C + D + E/5). The recognition threshold is expressed in the same manner. Higher scores indicate lower olfactory detection and recognition abilities.

All subjects were instructed to complete the Japanese version of the Montreal Cognitive Assessment (MoCA-J) (Nasreddine et al., 2005) under the supervision of neurologists. The MoCA can detect MCI with greater sensitivity than the Japanese version of the Mini Mental State Examination (Folstein et al., 1975). In this study, the MoCA was adapted for measurement of cognitive function.

### Imaging Data Acquisition

Magnetic resonance imaging (MRI) was performed at Ebara Hospital using a 3 Tesla MAGNETOM A Trio Tim Scanner (Siemens, Erlangen, Germany) with a 32-channel phased-array coil. The anatomical scan was acquired with a T1-weighted 3D MPRAGE sequence with the following parameters: 9 degree flip angle, TR 2300 ms, TE 2.98 ms, inversion time 900 ms, matrix size 256 × 256, field of view 256 mm and 176 slices with a voxel size of 1 mm^3^. T1-weighted images of all subjects were acquired with the same scanner at Ebara Hospital.

### Image Processing

Images were processed using FreeSurfer (Version 6) automated neuroanatomical segmentation software^2^. Gray matter volumes and cortical thicknesses were determined with FreeSurfer using the recon-all script on each T1-weighted scan. FreeSurfer processing includes motion correction, removing non-brain tissue, non-uniform intensity normalization, affine registration to Montreal Neurological Institute (MNI) space, Talairach transformation, volumetric segmentation (Fischl et al., 2002), cortical surface reconstruction (Dale et al., 1999; Fischl et al., 1999; Fischl and Dale, 2000), and parcelation (Fischl et al., 2004; Desikan et al., 2006). After affine registration to MNI space, all boundaries were visually inspected with a graphical viewer (Freeview and TkMedit with FreeSurfer). All volumetric segmentations were inspected for accuracy, and minor manual editing was performed by two trained neurologists with the TkMedit editing tool, and re-analyzed with recon-all script. Manual editing was restricted to removal of non-brain tissue included within the cortical boundary. The thickness of each ROI in the FreeSurfer atlas was obtained as output in *aparc.stats* files. The volume of each ROI was obtained from FreeSurfer output *aseg.stats* files and *aparc.stats* files. Because the intracranial volume (ICV) is an important covariate for volumetric analyses of the brain, ICV measurements obtained with FreeSurfer were confirmed with those measured with the Statistical Parametric Mapping 12 (SPM12) program (Ashburner and Friston, 2005). The ICV was calculated in SPM from each processed T1-weighted image using the Segment implementation tool (Ashburner and Friston, 2005) included in SPM12.

### Data Analysis

All data from FreeSurfer were entered into SPSS Statistics (IBM SPSS Statistics, Version 23.0, IBM Corp., Armonk, NY, United States) including age, sex, years of education, olfactory scores and MoCA scores, and all statistical analyses were performed using SPSS. ROIs specifically associated with olfaction including the AMG, HI, para-HI, OFC, MFC, and rectus were analyzed. The AMG, HI, and para-HI were selected as ROIs based on the results of a previous study (Watanabe et al., 2018) where they were found to play key roles in olfactory memory and odor-induced emotional responses. In addition to these areas, the para-HI, MFC and rectus were examined, based on studies suggesting that these areas are involved in conscious awareness of olfactory stimuli (Delon-Martin et al., 2013; Watanabe et al., 2018).

Group differences in age, years of education, MoCA-J score, olfactory detection and olfactory recognition levels were examined using the non-parametric Mann-Whitney test. Group comparisons for ICV and whole brain volume (WBV) were conducted using analysis of covariance (ANCOVA) with sex and years of education as covariates. Between-group differences in the left and right HI, AMG, OFC, para-HI, rectus, and MFC volumes were assessed using ANCOVA, with sex, years of education and ICV according to FreeSurfer (FreeSurfer ICV) included as covariates. To determine whether a differential relationship existed between olfactory recognition ability and volume of ROIs between groups, a general linear model was conducted. Each model included the main effects of group and volume and group × volume interactions, with FreeSurfer ICV and years of education as covariates. The main effects of group × volume interactions were the outcomes of interest.

To confirm accuracy of ICV measurement, the FreeSurfer ICV and the ICV measured with SPM (SPM ICV) were compared using a non-parametric Mann-Whitney *U* test. Regression analysis was performed to determine the correlation coefficient. Reanalysis using the SPM ICV was performed for all comparisons. Comparison of the SPM ICV between elderly subjects and young HCs was conducted by ANCOVA with sex and years of education as covariates. Between-group differences in the left and right HI, AMG, OFC, para-HI, rectus, and MFC volumes were assessed using ANCOVA, with sex, years of education and SPM ICV as covariates. General linear model was conducted with SPM ICV and years of education as covariates. A group comparison of cortical thickness was performed with ANCOVA with sex and years of education as covariates. The ICV was not included in the thickness analysis as a covariate.

Path analysis was conducted to examine how the ROIs impacted olfactory recognition and the MoCA in both groups. Path analysis is closely related to multiple regression. This analysis can be conducted to provide estimates of the magnitude and significance of hypothesized causal connections between sets of variables. The method utilizes information provided by the statistical correlations in conjunction with qualitative information regarding the causal relationships to identify the consequences of hypothesized associations. An advantage of using path analysis is that the effect of the indirect path (for example, whether the para-HI volume affects olfactory ability mediated through the HI) and the total effect can be determined (for example, the total effect on olfactory ability of both the HI and para-HI). The structural equation modeling program employed (IBM SPSS Statistics Amos, Version 23.0, IBM Corp., Armonk, NY, United States) specified statistical significance for path coefficients. Before interpreting the results from the path analysis, we carried out a number of checks to ensure the validity of the models using the goodness of fit index (GFI) and Bollen-Stine bootstrapping. A GFI close to 1 and Bollen-Stine bootstrap *P* value > 0.05 were adopted for the model. Before undertaking path analysis, partial correlation testing was performed to refine our hypotheses. Within-group partial correlation tests were conducted between olfactory ability or the MoCA score and each brain region volume, covarying with differences in age, years of education and sex. The results were corrected for multiple comparisons with a false discovery rate (FDR) of *P* < 0.05 (Benjamini and Hochberg, 1995).

## Results

### Demographic Results

Demographic data are displayed in Table 1. There were significant differences in age (*z* = -6.3, *P* < 0.001), years of education (*z* = -4.4, *P* < 0.001) and MoCA-J scores (*z* = -4.6, *P* < 0.001). Elderly subjects had significantly lower olfactory detection ability than young HCs (*z* = -5.1, *P* < 0.001). Five of 27 elderly subjects displayed impaired olfactory recognition, and the remaining 22 subjects also had lower olfactory recognition abilities (*z* = -4.4, *P* < 0.001; the average results from 22 subjects are indicated in Table 1). Table 1 shows a comparison of each volume between elderly subjects and younger HCs. Elderly subjects had a lower ICV (*F* = 8.53, *P* < 0.01, partial eta squared: ηp^2^, 0.15) and WBV (*F* = 41.46, *P* < 0.0001, ηp^2^, 0.45) and lower volumes of the left HI (*F* = 15.01, *P* < 0.0001, ηp^2^, 0.23), right HI (*F* = 11.5, *P* < 0.001, ηp^2^, 0.19), left AMG (*F* = 12.45, *P* < 0.001, ηp^2^, 0.21), right AMG (*F* = 12.66, *P* < 0.001, ηp^2^, 0.21), left OFC (*F* = 16.28, *P* < 0.0001, 0.25), right OFC (*F* = 13.76, *P* < 0.001, ηp^2^, 0.22), left MFC (*F* = 12.43, *P* < 0.001, ηp^2^, 0.2), and right MFC (*F* = 35.12, *P* < 0.0001, ηp^2^, 0.42) than young HCs. There were no significant differences in the left para-HI (*F* = 0.02, *P* = 0.86, ηp^2^, 0.001), right-para-HI (*F* = 0.86, *P* = 0.35, ηp^2^, 0.02), left rectus (*F* = 0.006, *P* = 0.94, ηp^2^, 0.0001), or right rectus (*F* = 3.3, *P* = 0.08, ηp^2^, 0.06) between the elderly subjects and young HCs.

Table 2 shows comparisons of the OFC, para-HI, rectus and MFC thickness between the two groups. No correlation was observed between thickness of an ROI and ICV (FreeSurfer and SPM) (Supplementary Table 1); therefore, ICV was not used as covariate for analysis of cortical thickness comparisons. Similar results to the volume comparison were observed for cortical thickness. There was no significant difference in the left para-HI (*F* = 1.23, *P* = 0.27, ηp^2^, 0.02), right para-HI (*F* = 0.001, *P* = 0.98, ηp^2^, 0.001), and left rectus (*F* = 0.91, *P* = 0.34, ηp^2^, 0.02) between groups. Significant decreases in the left OFC (*F* = 17.5, *P* = 0.001, ηp^2^, 0.26), right OFC (*F* = 7.87, *P* = 0.007), left MFC (*F* = 23.3, *P* = 0.001, ηp^2^, 0.32), right MFC (*F* = 14.7, *P* = 0.001, ηp^2^, 0.27), and right rectus (*F* = 5.26, *P* = 0.02, ηp^2^, 0.09) were observed in elderly subjects compared with young HCs.

**TABLE 2:** Comparison of cortical thickness between young HC and elderly subjects.

	Younger HC (24–55)	Elderly Subjects (62–84)
L-OFC (mm)	2.75 ± 0.13	2.59 ± 0.12***
R-OFC (mm)	2.8 ± 0.14	2.7 ± 0.14**
L-paraHI (mm)	3.07 ± 0.25	3.06 ± 0.29
R-paraHI (mm)	3.22 ± 0.2	3.16 ± 0.26
L-rectus (mm)	2.59 ± 0.14	2.55 ± 0.13
R-rectus (mm)	2.59 ± 0.18	2.45 ± 0.14*
L-MFC (mm)	2.67 ± 0.12	2.49 ± 0.11***
R-MFC (mm)	2.6 ± 0.09	2.49 ± 0.1***

*P < 0.05, **P < 0.01, ***P < 0.001.

### Interaction Between Volumes of ROIs and Groups

**FIGURE 1:**
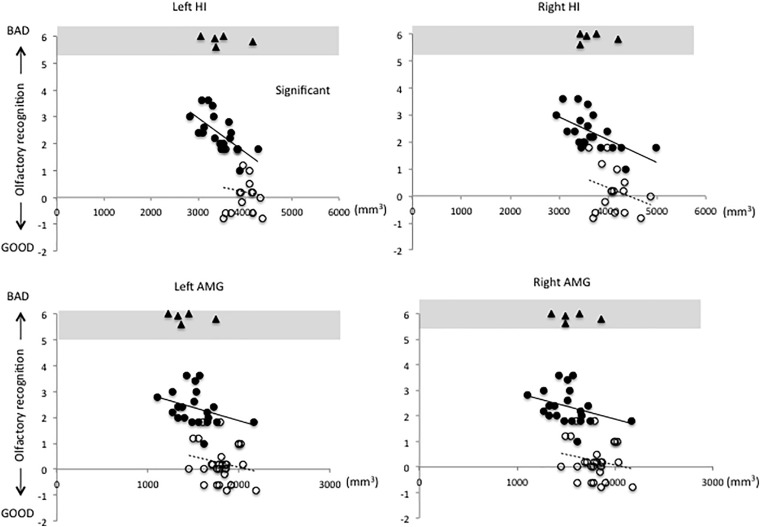
Relationships between volumes of the hippocampus (HI) and amygdala (AMG) and olfactory test scores in elderly and young subjects (elderly subjects, closed circles with solid line; young subjects, open circles with dotted line). A significant group × volume interaction was observed for the left HI. There were no group × volume interactions for any other brain regions (statistical details are included in Supplementary Table 2).

**FIGURE 2:**
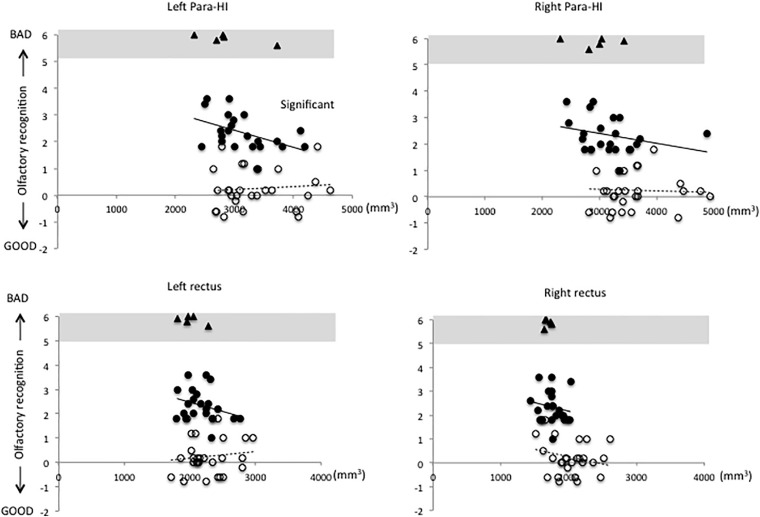
Relationships between volumes of the parahippocampus (para-HI) and rectus and olfactory test scores in elderly and young subjects. A significant group × volume interaction was observed for the left para-HI.

**FIGURE 3:**
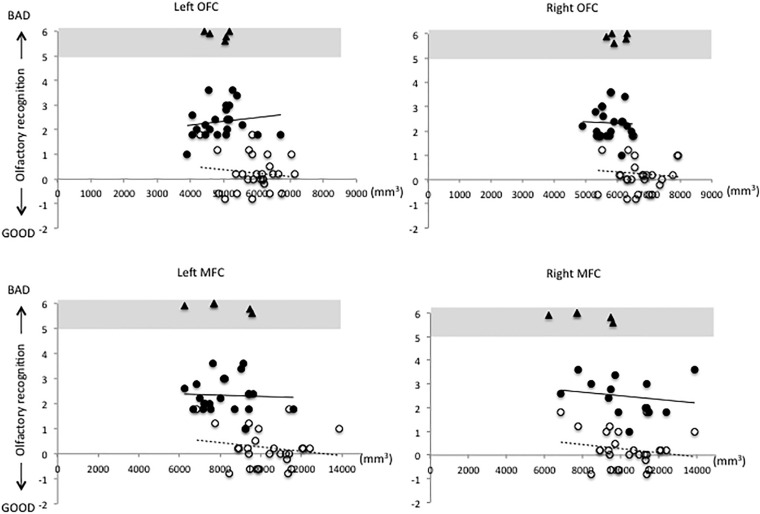
Relationships between volumes of the olfactory cortex (OFC) and medial prefrontal cortex (MFC) and olfactory test scores in elderly and young subjects.

Figures 1–3 show the relationship between volume and olfactory recognition levels in the elderly subjects (closed circles with a solid line) and young HCs (open circles with a dotted line). Five elderly subjects with impaired olfactory recognition were not included in the analysis, but data are included and highlighted in the upper region of each figure for reference. Significant group × volume interactions were found for the left HI (*P* = 0.005) and para-HI (*P* = 0.02). There were no group × volume interactions for other brain areas (detailed results are provided in Supplementary Table 2).

A general linear model was used to examine the effect of thickness and the group × cortical thickness interaction on olfactory recognition in elderly subjects and young HCs. A significant group × volume interaction was found for the left para-HI (*P* = 0.001) and right para-HI (*P* = 0.02) (Supplementary Figure 1, statistical details in Supplementary Table 3).

### Re-analysis Using the SPM ICV

The mean and standard deviation of the SPM ICV were 1445416 (mm^3^) @ 145802. The correlation coefficient of the FreeSurfer ICV and SPM ICV was 0.91 (*t* = 15.12, *P* = 0.0001, 95% CI, 0.82–1.07). Reliability test was performed between FreeSurfer ICV and SPM ICV (Cronbach’s alpha = 0.95). The estimated value was smaller for the SPM ICV than for the FreeSurfer ICV (*z* = −0.21, *P* = 0.03). Malone et al. (2015) reported that smaller ICV estimate with SPM12 might be caused by the blood-filled sinuses being effectively excluded from the tissue segments. This small bias need to concern when the ICV is used as a covariate for ANCOVA and analysis with linear models.

To confirm the FreeSurfer ICV results, re-analysis was performed using the SPM ICV. The results of group comparisons and the general linear model using the SPM ICV were consistent with those of the FreeSurfer ICV. In brief, a significantly lower SPM ICV was found in elderly subjects compared with young HCs (*F* = 7.77, *P* = 0.007, ηp^2^, 0.13) (Supplementary Table 4). A comparison of ROI volumes was performed with age, years of education and SPM ICV as covariates. Significant volume reductions were observed in the left and right HI, left and right AMG, left and right OFC, the right rectus, and left and right MFC. There were no differences in the left and right para-HI, and left and right rectus. Statistical details are indicated in Supplementary Table 5. General linear model was conducted with SPM ICV and years of education as covariates. A Significant group × volume interactions were found for the left HI (*P* = 0.001) and para-HI (*P* = 0.01). There were no group × volume interactions for other brain areas. The statistical details are indicated in Supplementary Table 6.

### Path Analysis

Because the path analysis included the HI and AMG volume, we considered that the same unit (mm^3^) could be used to help to understand the comparison. We focused on volume changes in the following analysis.

**FIGURE 4:**
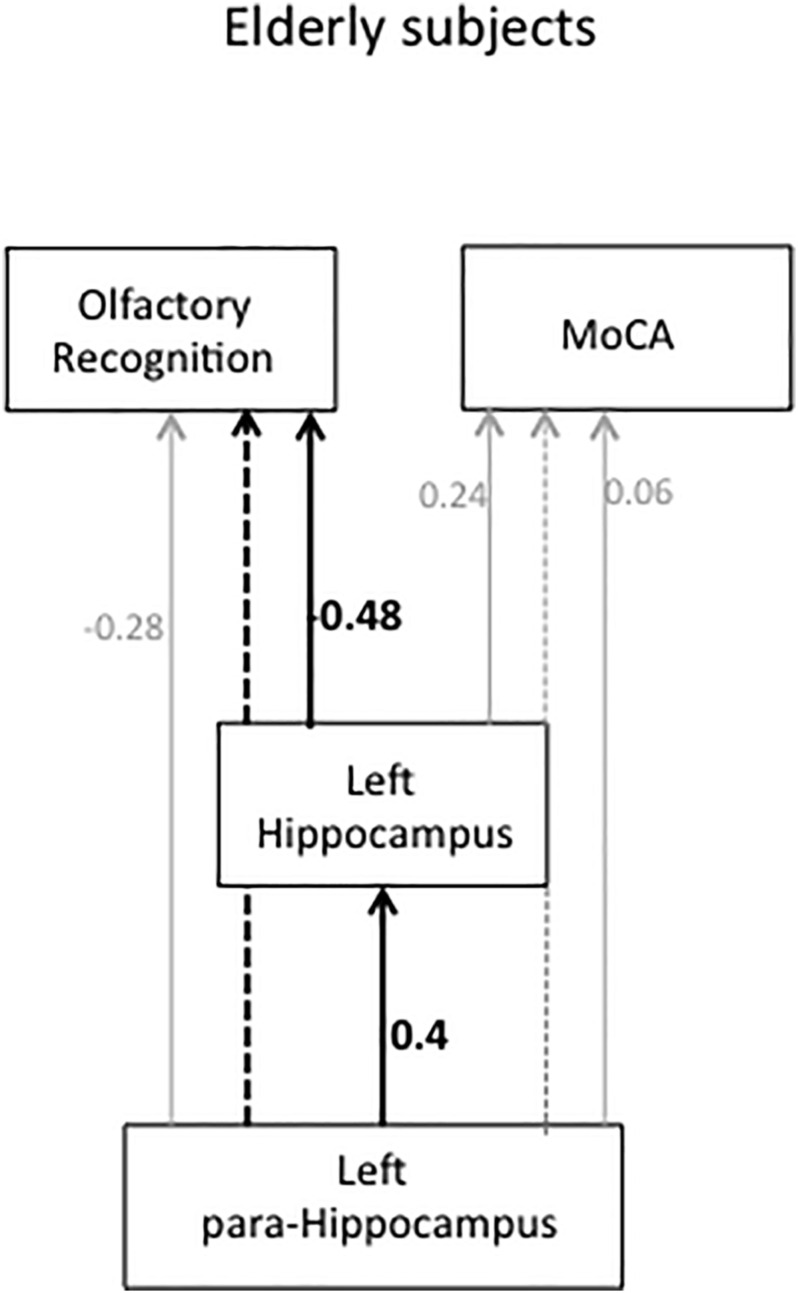
Path diagram and standardized path coefficients for elderly subjects. The solid lines indicate a significant direct effect. The dotted line indicates a significant indirect effect. Gray solid lines indicate a non-significant direct path, and the gray dotted line indicates a non-significant indirect path.

Before undertaking the path analysis, for exploratory analysis, partial correlation tests were performed within the groups. The details of these statistical results are provided in Supplementary Tables 7,8. In brief, there was a negative correlation between the olfactory recognition score and the left HI volume (*r* = −0.58, *P* = −0.008) and right HI volume (*r* = −0.45, *P* = 0.05) and between the olfactory score and the left para-HI volume (*r* = −0.45, *P* = −0.05). These correlations did not survive FDR correction (*P* > 0.05). No partial correlations were observed in young HCs (Supplementary Table 8). Path analyses were used to investigate how volumes of each brain region interacted in predicting MoCA and olfactory recognition scores for each group. We first tested the full model, including volumes of all ROIs and MoCA and olfactory recognition scores. The final path model was constructed by successively eliminating non-significant paths (GFI = 0.99, Bollen-Stine bootstrap *P* = 0.81). Figure 4 shows the path diagram and direct and indirect effects of standardized path coefficients for elderly subjects (GFI = 0.99).

The left HI had a direct path to olfactory recognition (*r* = −0.48, *P* = 0.009), indicating that decreased olfactory ability was associated with a smaller left HI volume. Smaller HI volume was predicted by a smaller left para-HI volume (*r* = 0.4, *P* = 0.04). A smaller left para-HI volume indirectly affected decreased olfactory recognition mediated through the left HI (*P* = 0.005). The left para-HI volume had no direct effect on olfactory recognition (*r* = −0.28, *P* = 0.15). There was a significant total effect (direct + indirect) of para-HI volume on olfactory recognition (*P* = 0.005). Left HI and left para-HI volume had no direct effect on the MoCA (*r* = 0.24, *P* = 0.31, *r* = 0.06, *P* = 0.81, respectively). There was no indirect effect from the para-HI on the MoCA through the left HI (*P* = 0.21). Path analyses were performed for the young HCs in the same manner, and no significant paths were found (*P* > 0.05). Detailed statistical values for indirect and total effects are indicated in Supplementary Table 9.

## Discussion

Our specific interest was to determine which olfactory areas were related to the decline of olfactory ability in elderly subjects who self-reported no cognitive impairment. Our aim was to test whether morphological changes might be related to the changes in olfactory ability that occur before cognitive decline. The most interesting findings in this study were observed in path analysis, indicating that direct and indirect effects of HI and para-HI volume on olfactory ability. Volume reductions in these areas were not associated with cognitive function measured by the MoCA. This means that subjects with smaller HI and para-HI volumes presented with impaired olfactory abilities prior to the decline of cognitive function.

### The HI and Para-HI Are Associated With Olfactory Decline

Overall brain volume reductions were observed in elderly subjects compared with young HCs, but only reduction in the volume of the left HI was associated with decreased olfactory ability. On the one hand, it was interesting to observe that the volume of the para-HI in elderly subjects was not different from that of young HCs; on the other hand, the extent of the reduction of para-HI volume varied among individuals, and this reduction was associated with olfactory decline. Together, these findings suggest that the association between left HI and left para-HI volume changes might play a key role in olfactory recognition.

Path analysis indicated that HI volume reduction directly affects olfactory decline and that the para-HI is indirectly impacted by reducing olfaction levels through the left HI. A limitation of the study is that this model does not indicate cause and effect; therefore, it could not be determined whether left HI volume might be reduced prior to the left para-HI volume or vice versa. This leaves open the question of whether olfactory impairment may be caused be pathological changes in the HI or para-HI. We speculate that the para-HI might be the first region associated with olfactory recognition to undergo damage. This would be consistent with the pathological changes that begin in the ENT and para-HI in the early stages of AD (Koychev et al., 2017). Indeed, a previous study found that patients with moderate AD had intact olfactory detection levels, but olfactory recognition was specifically impaired (Masaoka et al., 2013). The ability to identify odors requires memory retrieval and labeling the name of the odor and may specifically require the functions of both the HI and OFC. In Parkinson’s disease, the impairment of olfactory ability has been suggested to result from disconnection between the HI and OFC (Masaoka et al., 2007).

In general, the para-HI plays an important role in memory retrieval and recollection of memory and is a key area for processing cognitive context (Naya, 2016). The para-HI is involved in processing memory of “where” events and is associated with the spatial contextual information of episodic memory (Eichenbaum et al., 2007). The para-HI is activated in parallel with the activity of the posterior OFC observed in odor-induced autobiographical memory (Watanabe et al., 2018), suggesting that the para-HI may function in relaying past memories and identification or recognition of memories. Anatomically, the para-HI is part of the medial temporal region, and the anterior part of the para-HI is closest to the ENT, which is the gateway to the dentate gyrus of the HI (Eichenbaum et al., 2007). The perirhinal cortex and para-HI have bi-directional projections to the AMG, OFC, cingulate gyrus and parietal cortex (Suzuki and Naya, 2014; Naya, 2016). The para-HI may have important roles in relaying memory retrieval and emotional reaction to conscious awareness of the context organized in the OFC. It is possible that volume changes of the HI and para-HI caused by pathological changes might contribute to the decline of olfactory recognition, where they play important roles in the relay areas projecting to the OFC.

Olfactory decline was associated with HI and para-HI volume changes in this study, especially in the left hemisphere. Left HI volume reduction associated with memory and cognitive function has been reported at the onset of AD (Peng et al., 2015) and psychiatric disorders (Velakoulis et al., 1999; Wannan et al., 2019). It is unknown why the left medial temporal regions are damaged prior to the onset of typical disease symptoms. Further research in a longitudinal study could help to determine whether current findings are relevant to MCI and AD patients.

### Volume Reduction and Cortical Thinning

In our study, similar results to the volume comparison were observed for cortical thickness. However, the right rectus thinning was observed in elderly subjects. Volume reduction of the right rectus did not reach significant although having trends (*P* = 0.06 and 0.07, FreeSurfer ICV and SPM ICV, respectively). Additionally, significant group × thickness interaction was found for right para-HI (general linear model, Supplementary Table 3) as well as the left para-HI. It could be possible to assume that cortical thinning might be more sensitive to detect brain change than the volume reduction. The right rectus and right para-HI were not survived for the results of the path analysis, however, a relation between cortical thinning and volume reduction was interesting theme for understanding brain pathology.

Storsve et al. (2014) reported the importance of longitudinal studies for determining changes in cortical thickness, surface area and volume with aging. They reported that volume changes were closely related to thickness changes and found that area and thickness changes appeared to have both overlapping and different effects on volume across the adult life span (Storsve et al., 2014). The areas that first show cortical thinning or volume reductions with age are worth investigating.

In addition, in our study smaller ICV was observed in elderly subjects. Regarding ICV reduction in elderly subjects, age-related volumetric decline progresses by the age of 75 years (Barnes et al., 2010). It is unknown how the amount that cortical thinning or volume reduction may primarily contribute to ICV volume changes. Storsve et al. (2014) reported a mixture of accelerating or decelerating brain changes with age. In our study, although brain volume reductions and cortical thinning were observed in most regions, there was no link to cognitive function and olfactory ability. On the other hand, para-HI volume reduction and thinning varied among individuals, and this small amount of individuality was reflected in olfactory impairment. It is not known how elderly subjects with olfactory decline progress to cognitive impairment, and whether the para-HI accelerate to reduce volume in the further stage. A longitudinal study is required to answer these questions.

### Limitations

Several other limitations of the current study should be mentioned. Our study was limited by a small sample size. Additional imaging data and olfactory tests in HCs as well as MCI and AD patients will be necessary to confirm our results. The HI consists of several functionally distinct regions, including the Cornus Ammonis (CA1–4), dentate gyrus, subiculum and stratum layers, and the dentate gyrus is an active region for generation of new neurons (Rietze et al., 2000). Of interest is how the ENT, perirhinal cortex and para-HI interact with the dentate gyrus of the HI, the only region where neurogenesis occurs in the brain, and the olfactory bulb (Kempermann et al., 2018). The relationships between olfactory ability and medial temporal sub-regions and hippocampal subfields also worthy of further investigation.

## Data Availability Statement

The raw data supporting the conclusions of this article will be made available by the authors, without undue reservation.

## Ethics Statement

The studies involving human participants were reviewed and approved by Ethical Committees of Showa University School of Medicine. The patients/participants provided their written informed consent to participate in this study.

## Author Contributions

SK and YM designed the study, conducted experiments, analyzed the MRI data, and wrote the initial manuscript. HS, SK, AY, and MH conducted and analyzed olfactory testing and the MoCA. SK, MY, AY, NK, MH, RK, KW, NI, and MI recruited participants, assisted with scanning, and performed pre-processing of MRI data. All authors discussed the results. YM, KO, and MIZ edited the final manuscript.

## Conflict of Interest

The authors declare that this study received funding from Kao Corporation. The funder had the following involvement with the study: HS was employed by Kao Corporation and was involved in collection and analysis of olfaction and MoCA data.

## Footnotes

1 https://www.wma.net/policies-post/wma-declaration-of-helsinki-ethical-principles-for-medical-research-involving-human-subjects/

2 http://surfer.nmr.mgh.harvard.edu
