# Supplementary material for: Hippocampus and Parahippocampus Volume Reduction Associated With Impaired Olfactory Abilities in Subjects Without Evidence of Cognitive Decline
Source: Front Hum Neurosci. 2020 Sep 30;14:556519. doi: 10.3389/fnhum.2020.556519 (PMC7556227; doi:10.3389/fnhum.2020.556519)
Supplement: Supplementary file 1 [file Table_1.DOC]

**5SUPPLEMENTAL MATERIALS**

**Hippocampus and parahippocampus volume reduction associated with impaired olfactory abilities prior to the decline of cognitive function**

Satomi Kubota^1,2^, Yuri Masaoka^1*^, Haruko Sugiyama^3^, Masaki Yoshida^4^, Akira Yoshikawa^1^, Nobuyoshi Koiwa^5^, Motoyasu Honma ^1^, Ryuta Kinno ^2^, Keiko Watanabe^2^, Natsuko Iizuka ^1,2^, Masahiro Ida^6^, Kenjiro Ono^2^, Masahiko Izumizaki ^1^

1. Department of Physiology, Showa University School of Medicine
2. Division of Neurology, Department of Medicine, Showa University School of Medicine
3. Sensory Science Research, Kao Corporation
4. Department of Ophthalmology, Jikei Medical University
5. Department of Health and Science, University of Human Arts and Sciences
6. National Hospital Organization Mito Medical Center

*Corresponding author

Yuri Masaoka

Department of Physiology, Showa University School of Medicine

1-5-8 Hatanodai, Shinagawa-ku, Tokyo 142-8555, Japan

Telephone: +81 3 3784 8113

Fax: +81 3 3784 0200

Mail: [faustus@med.showa-u.ac.jp](mailto:faustus@med.showa-u.ac.jp)

Supplemental Table 1. Correlation between cortical thickness and ICV in elderly subjects and young HCs.

| Elderly subjects | | L-OFC | R-OFC | L-paraHI | R-paraHI | L-rectus | R-rectus | L-MFC | R-MFC |
| --- | --- | --- | --- | --- | --- | --- | --- | --- | --- |
| FreeSurfer | Coefficient | -0.11 | -0.22 | -0.22 | -0.32 | 0.01 | -0.09 | -0.16 | -0.18 |
| ICV | P value | 0.56 | 0.26 | 0.25 | 0.10 | 0.95 | 0.62 | 0.40 | 0.35 |
| SPM ICV | Coefficient | -0.07 | -0.18 | -0.20 | -0.36 | 0.11 | -0.0 | -0.2 | -0.1 |
|  | P value | 0.70 | 0.36 | 0.30 | 0.06 | 0.56 | 0.69 | 0.30 | 0.60 |
| YoungHC |  |  |  |  |  |  |  |  |  |
| FreeSurfer | Coefficient | -0.04 | -0.2 | 0.06 | -0.08 | 0.26 | 0.16 | 0.05 | -0.02 |
| ICV | P value | 0.8 | 0.31 | 0.74 | 0.69 | 0.18 | 0.42 | 0.78 | 0.88 |
| SPM ICV | Coefficient | -0.172 | -0.32 | -0.11 | -0.22 | 0.11 | 0.27 | -0.18 | -0.27 |
|  | P value | 0.39 | 0.09 | 0.56 | 0.26 | 0.57 | 0.17 | 0.36 | 0.15 |

Supplemental Table 2. General linear model examining the effects of volume and group × volume interactions on olfactory recognition in elderly subjects and young HCs. Sex, years of education and FreeSurfer ICV as covariates.

|  | Volume | | | Group　×　Volume | | |
| --- | --- | --- | --- | --- | --- | --- |
|  | Wald *X*2 | Exp.B(95% CI) | p value | Wald X2 | Exp.B(95% CI) | p value |
|  |  |  |  |  |  |  |
| L-HI | 0.78 | 1.00(0.99-1.00) | 0.37 | 8.01 | 0.99(0.99-1.00) | 0.005** |
| R-HI | 0.02 | 1.00(0.99-1.00) | 0.88 | 1.81 | 0.99(0.99-1.00) | 0.17 |
| L-AMG | 0.18 | 1(0.99-1.00) | 0.66 | 0.25 | 0.99(0.99-1.00) | 0.61 |
| R-AMG | 1.61 | 0.99(0.99-1.00) | 0.2 | 0.05 | 1(0.99-1.00) | 0.82 |
| L-OFC | 0.21 | 1.00(0.99-1.00) | 0.64 | 0.31 | 1.00(0.99-1.00) | 0.57 |
| R-OFC | 0.23 | 1.00(0.99-1.00) | 0.62 | 0.02 | 1.00(0.99-1.00) | 0.87 |
| L-paraHI | 0.83 | 0.99(0.99-1.00) | 0.36 | 5.84 | 1.00(1.00-1.01) | 0.02* |
| R-paraHI | 0.1 | 1.00(0.99-1.00) | 0.75 | 1.4 | 1.00(0.99-1.00) | 0.23 |
| L-rectus | 0.17 | 0.99(0.99-1.00) | 0.67 | 3.63 | 0.99(0.99-1.00) | 0.06 |
| R-rectus | 0.68 | 0.99(0.99-1.00) | 0.41 | 0.09 | 0.99(0.99-1.00) | 0.76 |
| L-MFC | 0.71 | 1.00(1.00-1.00) | 0.39 | 0.05 | 1.00(1.00-1.00) | 0.81 |
| R-MFC | 0.74 | 1.00(1.00-1.00) | 0.39 | 0.66 | 1.00(1.00-1.00) | 0.41 |
|  |  |  |  |  |  |  |

**P<0.01, *P<0.05

Supplemental Table 3. General linear model examining the effects of thickness and group × cortical thickness interactions on olfactory recognition in elderly subjects and young HCs. Sex, years of education as covariates.

|  | Thickness | |  | Group　×　Thickness | |  |
| --- | --- | --- | --- | --- | --- | --- |
|  | Wald *X*2 | Exp.B(95% CI) | p value | Wald X2 | Exp.B(95% CI) | p value |
|  |  |  |  |  |  |  |
| L-OFC | 3.4 | 6.1(0.89-42.5) | 0.06 | 0.14 | 0.54(0.02-12.37) | 0.71 |
| R-OFC | 5.5 | 9.1(1.45-56.6) | 0.01 | 1.45 | 0.19(0.01-2.7) | 0.22 |
| L-paraHI | 6.1 | 3.5(1.2-9.3) | 0.01 | 10.55 | 0.11(0.03-0.42) | 0.001*** |
| R-paraHI | 3 | 3.48(0.85-14.15) | 0.08 | 5.37 | 0.12(0.02-0.72) | 0.02* |
| L-rectus | 13.9 | 23.3(4.46-122.1) | 0.0001 | 1.26 | 0.23(0.02-2.9) | 0.26 |
| R-rectus | 1.5 | 0.4(0.09-1.7) | 0.21 | 1.42 | 4.7(0.36-61.3) | 2.33 |
| L-MFC | 0.21 | 0.61(0.73-5.1) | 0.64 | 0.37 | 0.35(0.01-9.81) | 0.54 |
| R-MFC | 0.01 | 1.17(0.07-19.6) | 0.91 | 0.65 | 0.19(0.004-10.12) | 0.42 |

***P<0.001, *P<0.05

Supplemental Table 4. ICV measured with SPM was compared with elderly subjects and young HCs.

|  | Younger HC (24-55) | Elderly Subjects (62-84) | F | P | ηp^2^ |
| --- | --- | --- | --- | --- | --- |
| SPM ICV | 1508438 ± 135483 | 1382395 ± 129341 | 7.77 | 0.007 | 0.13 |

Supplemental Table 5. Comparison of each volume between elderly subjects and young HCs with

Sex, years of education and SPM ICV as covariates. Mean volume and standard deviations indicated same value in Table 1 on the article.

|  | Younger HC (24-55) | Elderly Subjects (62-84) | F | P | ηp^2^ |
| --- | --- | --- | --- | --- | --- |
| L-HI | 4023 ± 282 | 3472 ± 349 | 16.11 | 0.0001*** | 0.25 |
| R-HI | 4261 ± 360 | 3666 ± 443 | 12.52 | 0.001** | 0.21 |
| L-AMG | 1731 ± 176 | 1388 ± 228 | 12.15 | 0.001** | 0.2 |
| R-AMG | 1795 ± 171 | 1527 ± 214 | 12.11 | 0.001** | 0.2 |
| L-OFC | 5939 ± 674 | 4921 ± 613 | 16.94 | 0.0001*** | 0.26 |
| R-OFC | 6672 ± 670 | 5811 ± 402 | 14.08 | 0.0001*** | 0.22 |
| L-paraHI | 3350 ± 596 | 3096 ± 496 | 0.02 | 0.88 | 0.0001 |
| R-paraHI | 3590 ± 559 | 3129 ± 510 | 1.11 | 0.29 | 0.02 |
| L-rectus | 2271 ± 320 | 2151 ± 244 | 0.08 | 0.76 | 0.002 |
| R-rectus | 2015 ± 288 | 1753 ± 147 | 3.42 | 0.07 | 0.07 |
| L-MFC | 10288 ± 1539 | 8214 ± 1280 | 13.42 | 0.001** | 0.21 |
| R-MFC | 9309 ± 1307 | 7495 ± 814 | 36.4 | 0.0001*** | 0.42 |

*** P<0.0001, **P<0.001, *P<0.01

Supplemental Table 6. General linear model examining the effects of volume and group × volume interactions on olfactory recognition in elderly subjects and young HC. Sex, years of education and SPM ICV as covariates.

|  | Volume | | | Group　×　Volume | | |
| --- | --- | --- | --- | --- | --- | --- |
|  | Wald *X*2 | Exp.B (95% CI) | p value | Wald X2 | Exp.B(95% CI) | p value |
|  |  |  |  |  |  |  |
| L-HI | 2.48 | 1.00(1.00-1.00) | 0.11 | 10.34 | 0.99(0.99-0.99) | 0.001** |
| R-HI | 0.02 | 1.00(0.99-1.00) | 0.86 | 3.19 | 0.99(0.99-1.00) | 0.07 |
| L-AMG | 0.19 | 1.00(0.99-1.00) | 0.65 | 0.42 | 0.99(0.99-1.00) | 0.51 |
| R-AMG | 2.4 | 0.99(0.99-1.00) | 0.14 | 0.14 | 1.00(0.99-1.00) | 0.71 |
| L-OFC | 0.39 | 1.00(0.99-1.00) | 0.53 | 0.41 | 1.00(0.99-1.00) | 0.52 |
| R-OFC | 0.34 | 1.00(0.99-1.00) | 0.56 | 0.03 | 1.00(0.99-1.00) | 0.86 |
| L-paraHI | 1.19 | 1.00(1.00-1.00) | 0.27 | 6.09 | 0.99(0.99-1.01) | 0.01* |
| R-paraHI | 0.21 | 1.00(1.00-1.00) | 0.64 | 1.54 | 1.00(0.99-1.00) | 0.21 |
| L-rectus | 0.25 | 1.00(0.99-1.00) | 0.61 | 2.48 | 0.99(0.99-1.00) | 0.11 |
| R-rectus | 0.87 | 1.00(0.99-1.00) | 0.34 | 0.09 | 1.00(0.99-1.00) | 0.76 |
| L-MFC | 0.92 | 1.00(1.00-1.00) | 0.33 | 0.05 | 1.00(1.00-1.00) | 0.81 |
| R-MFC | 1.19 | 1.00(1.00-1.00) | 0.16 | 0.74 | 1.00(1.00-1.00) | 0.38 |

**P<0.001, *P<0.05

Supplemental Table 7. Partial correlation between MoCA, olfactory detection, olfactory recognition and volumes of olfactory ROI covarying with differences in age, years of education and sex in elderly subjects.

|  | MoCA-J | | Olfactory detection | | Olfactory recognition | |
| --- | --- | --- | --- | --- | --- | --- |
| Variables | Coefficient | P value | Coefficient | P value | Coefficient | P value |
| Olfactory detection | 0.18 | 0.44 | - | - | - | - |
| Olfactory recognition | -0.2 | 0.35 | 0.14 | 0.56 | - | - |
|  |  |  |  |  |  |  |
| L-HI | 0.32 | 0.17 | -0.24 | 0.32 | -0.58 | 0.008 * |
| R-HI | 0.31 | 0.20 | -0.43 | 0.06 | -0.45 | 0.05 * |
| L-AMG | 0.18 | 0.45 | -0.02 | 0.91 | -0.20 | 0.40 |
| R-AMG | 0.21 | 0.39 | -0.08 | 0.74 | -0.08 | 0.73 |
| L-OFC | -0.02 | 0.91 | 0.11 | 0.63 | 0.36 | 0.12 |
| R-OFC | -0.05 | 0.81 | -0.14 | 0.56 | 0.28 | 0.23 |
| L-paraHI | 0.07 | 0.77 | -0.27 | 0.25 | -0.45 | 0.05 * |
| R-paraHI | 0.01 | 0.94 | -0.14 | 0.55 | -0.03 | 0.87 |
| L-rectus | 0.02 | 0.93 | -0.15 | 0.51 | -0.11 | 0.63 |
| R-rectus | -0.24 | 0.31 | 0.11 | 0.63 | 0.02 | 0.91 |
| L-MFC | 0.02 | 0.92 | -0.13 | 0.57 | 0.04 | 0.86 |
| R-MFC | 0.10 | 0.67 | 0.01 | 0.94 | 0.07 | 0.77 |

*P<0.05, **P<0.01

Supplemental Table 8. Partial correlation between MoCA, olfactory detection, olfactory recognition and volumes of olfactory ROI covarying with differences in age, years of education and sex in young HCs.

|  | MoCA-J | | Olfactory detection | | Olfactory recognition | |
| --- | --- | --- | --- | --- | --- | --- |
| Variables | Coefficient | P value | Coefficient | P value | Coefficient | P value |
| Olfactory detection | -0.24 | 0.24 | - | - | - | - |
| Olfactory recognition | 0.25 | 0.22 | 0.71 | 0.0001 | - | - |
|  |  |  |  |  |  |  |
| L-HI | 0.18 | 0.37 | 0.09 | 0.65 | 0.16 | 0.42 |
| R-HI | -0.03 | 0.86 | 0.15 | 0.45 | -0.02 | 0.92 |
| L-AMG | 0.03 | 0.85 | -0.07 | 0.72 | -0.06 | 0.75 |
| R-AMG | -0.38 | 0.06 | 0.03 | 0.86 | -0.21 | 0.29 |
| L-OFC | 0.13 | 0.53 | -0.21 | 0.31 | -0.05 | 0.81 |
| R-OFC | 0.05 | 0.79 | -0.1 | 0.62 | -0.02 | 0.91 |
| L-paraHI | 0.29 | 0.15 | 0.07 | 0.72 | 0.15 | 0.45 |
| R-paraHI | 0.39 | 0.05* | -0.17 | 0.39 | -0.01 | 0.97 |
| L-rectus | -0.02 | 0.89 | -0.04 | 0.83 | 0.16 | 0.44 |
| R-rectus | -0.02 | 0.92 | -0.15 | 0.46 | -0.11 | 0.58 |
| L-MFC | -0.12 | 0.56 | -0.08 | 0.68 | -0.07 | 0.71 |
| R-MFC | -0.22 | 0.27 | -0.15 | 0.45 | -0.16 | 0.42 |

*P<0.05

|  |  |  |  |  |  |  |
| --- | --- | --- | --- | --- | --- | --- |

Supplemental Table 9. Standardized indirect and total effects with lower and upper bound limits.

| Bootstrapping (95% C) for the indirect effect | | |  |  |  |
| --- | --- | --- | --- | --- | --- |
|  |  |  | Lower bound | Upper bound | Sig. |
| L-paraHI | → | MoCA | -0.17 | 0.34 | 0.21 |
|  |  |  |  |  |  |
| L-para-HI | → | Olfactory Recognition | -0.38 | -0.04 | 0.005 |
|  |  |  |  |  |  |
|  |  |  |  |  |  |
| Bootstrapping (95% C) for the total effect | | |  |  |  |
|  |  |  |  |  |  |
| L-paraHI | → | MoCA | -0.21 | 0.57 | 0.44 |
|  |  |  |  |  |  |
| L-para-HI | → | Olfactory Recognition | -0.71 | -0.12 | 0.005 |
|  |  |  |  |  |  |

Supplemental Figure 1. Relationships between thickness of left para-hippocampus and olfactory test scores in elderly and young subjects (elderly subjects, closed circles with solid line; young subjects, open circles with dotted line). A significant group × thickness interaction was observed for the left and right para-hippocmapus. There were no group × thickenss interactions for any other brain regions (statistical details are included in Supplemental Table 3).
